# Novel Perspective of Cardiovascular Diseases: Volume-Regulatory Anion Channels in the Cell Membrane

**DOI:** 10.3390/membranes12070644

**Published:** 2022-06-23

**Authors:** Liming Hou, Yan Liu, Chao Sun, Rong Xu, Guihua Cao, Xiaoming Wang

**Affiliations:** Department of Geriatrics, Xijing Hospital, Fourth Military Medical University, Xi’an 710032, China; houlm11@163.com (L.H.); liuyanjelly@163.com (Y.L.); sjq20180620@163.com (C.S.); xurongkeke@126.com (R.X.); drcaoguihua@163.com (G.C.)

**Keywords:** volume-regulated anion channel (VRAC), cardiovascular disease, review

## Abstract

Cardiovascular diseases (CVDs) are the leading cause of morbidity and mortality worldwide. Although there are established mechanisms and preventions for CVDs, they are not totally elucidative and effective. Emerging evidence suggests that the dysregulation of ion channels in the cell membranes underpins the dysfunction of the cardiovascular system. To date, a variety of cation channels have been widely recognized as important targets for the treatment of CVDs. As a critical component of the anion channels, the volume-regulated anion channel (VRAC) is involved in a series of cell functions by the volume regulation and maintenance of membrane homeostasis. It has been confirmed to play crucial roles in cell action potential generation, cell proliferation, differentiation and apoptosis, and the VRAC appears to be a major participant in metabolic processes during CVDs. This review summarizes the current evidence and progress concerning the VRAC, to determine the future directions and challenges for CVDs for both preventive and therapeutic purposes.

## 1. Introduction

A total of 54.5 million deaths occurred globally in 2019, of which CVDs were responsible for 17.9 million cases (32%); CVDs are the leading cause of premature death around the world, and based on the data from the WHO, half a billion people have been afflicted by CVDs [1]. Thus, CVDs constitute a huge burden to the healthcare system, so it is particularly important to explore new targets for the prevention and treatment of CVDs.

Activated in response to an increase in cell volume and the condition of the outflow of chlorides and organic osmolytes from the cell, the VRAC is now known to be ubiquitously expressed in mammalian cells, including kidney, gastrointestinal tract, cholangiocytes and cardiovascular system [2]. In recent years, the VRAC has attracted the attention of researchers, hence a close relationship between the VRAC and cardiovascular diseases has been established. The VRAC modulates myocardial injury, ischemic preconditioning, and apoptosis, contributing to cardiovascular diseases including cardiac hypertrophy, atrial fibrillation and vascular remodeling during hypertension. Given these findings, the cardiac VRAC represents an important novel target for therapeutic approaches to cardiac diseases [3].

Therefore, the aim of this review is to summarize the structure and the functions of the VRAC, as well as the potential role of VRAC in regulating the pathophysiology of the cardiovascular system, to determine the future direction and challenges for both prevention and therapeutic purposes. 

## 2. Biophysical and Pharmacological Properties of VRAC

### 2.1. Molecular Structure of VRAC 

In 1978, Ehrlich ascites tumor cells and human lymphocytes were found to express the channel as an inward current triggered by osmotic swelling, thus influencing anion permeability [4]. Shortly thereafter, the comprehensive biophysical analysis of the VRAC was initiated, several candidates were considered as expression forms of the VRAC, including p-glycoprotein, pI_Cln_, phospholemman, voltage-gated chloride channels-2/3(CLC-2/3), bestrophins and the transmembrane protein 16(TMEM16)/anoctamin channel family, but they were all shown to be unnecessary for the VRAC or biophysical properties incompatible with VRAC later. Until 2014, the Jentsch group and Ardem Patapoutian’s laboratory simultaneously reported that the VRAC was composed of leucine-rich repeat containing 8 family members (LRRC8, LRRC8A-LRRC8E). LRRC8 proteins form the VRAC channel pore and are composed of two main parts: N-terminal 14 transmembrane helices (TM) and C-terminal 17 leucine-rich repeats (LRRs). LRRC8A is a necessary core component of VRAC activity, also known as Swell1. It has been confirmed that LRRC8A is widely distributed and expressed in the membranes of tissues and cell types. Mediated by the LRRs domain, it may exhibit multiple functions through protein interactions, which have now provided a firm basis for clarifying many burning questions in the field of cell volume regulation [5,6].

### 2.2. Physiological Characteristics of VRAC

So far, the VRAC had been reported playing a wide range of important physiological roles, including, (1) electrogenesis. Activation of the VRAC contributes to a mild depolarization of the resting potential and shortening of action potential duration in mammalian cells. (2) Cell proliferation. It appears to depend on functional Cl^−^ channels, inhibition of the VRAC suppresses cell proliferation in many cell types. (3) Cell differentiation. It seems clear that there is an association between the expression of VRAC and the process of transformation of cervical epithelial cells, muscle cells, microglia. (4) Regulated volume reduction (RVD) is more dependent on swell-activated Cl^−^ efflux (I_Cl,swell_) mediated by the VRAC channels; the VRAC is activated by osmotic swelling stimulation that involves sensitivity of ionic strength and membrane tension. (5) Apoptosis. VRAC activation is involved in apoptosis by its implication in apoptotic volume decrease phenomenon, and had been shown in Jurka cells, hepatoma and rat hepatocytes [7]. Furthermore, the membrane cholesterol content affects the equilibrium between the closed and open states of the VRAC channel rather than the basic pore properties of the channel, and the changes in membrane cholesterol modulates VRAC activity by affecting the membrane deformation energy associated with channel opening [8].

Different from other types of chlorine channels, the VRAC showed specific electrophysiological characteristics which are an outward rectification current activated slowly when cells are under hypotonic stimulation for 2~3 min, and the currents are inhibited in a hypertonic environment when cell volume shrinks, and exhibit a permeability in the order of SCN^−^ > I^−^ > NO_3_^−^ > Br^−^ > Cl^−^ > HCO_3_^−^ > glycine > F^−^ > taurine > lactate > gluconate > glutamate > aspartate [9]. Therefore, it is also called a volume-sensitive organic osmolyte/anion channel (VSOAC). In addition, the VRAC appeared to influence the multidrug resistance of cancer cells through the appropriate diameter concerning small-molecule medicinal substances and constituent proteins mediating the signaling pathways that modulate drug-induced cell death and facilitate AVD-related apoptosis [10].

### 2.3. Pharmacology of VRAC

The pharmacology of the chloride channel is quite disappointing compared with cationic channels. Although there are no fully specific VRAC inhibitors available, a number of compounds had been proved to strongly or partially inhibit the VRAC current, including 4,4′-diisothiocyanostilbene-2,2′-disulfonic acid (DIDS), 5-Nitro-2-(3-phenylpropylamino)-benzoic acid (NPPB), and tamoxifen, but all lack specificity. Endovion was found to be a voltage-independent, reversible blocker of VRAC with the highest affinity of any VRAC inhibitor yet tested (IC_50_ = 0.4 μM), but this compound has an off-target effect on calcium-activated Cl^−^ channels. It was found to inhibit calcium-activated Cl^−^channels (CaCCs) in ELA cells with an estimated IC_50_ of around 1.5 μM at negative potentials [11], Sauter et al. found that endovion altered ATP-induced [Ca^2+^]_i_ signals in the cells [12]. In vitro, natural phenol-phloretin inhibited VRAC in a voltage-independent and reversible manner with an IC50 of 30 μM. However, phloretin also inhibited CFTR and a variety of cation channels. Because the inhibitory effect of 4-(2-butyl-6,7-dichlor-2-cyclopentylindan-1-on-5-yl (DCPIB) on VRAC was significantly better than that of cystic fibrosis transmembrane conductance regulator (CFTR), calcium-activated chloride channels (CaCCs), CLCs and other chloride channels, and it was verified in vascular endothelial cells, cardiomyocytes and other cells (IC_50_, 2~5 μM), DCPIB has become the most commonly used and promising VRAC blocker. In addition, David presented that in lipid nanodiscs, DCPIB plugs the VRAC like a cork in a bottle, binding into the extracellular selectivity filter and sterically occluding ion conduction, and the lipid bilayers demonstrated a critical role in determining the VRAC structure [13]. In 2019, Eric et al. discovered the cysteinyl leukotriene receptor 1 (CysLT1R) antagonist, pranlukast and zafirlukast as novel inhibitors of endogenous VRAC [14].

In addition, zinc pyridinium thione (ZPT) was found to activate the VRAC in HEK293 cells with an EC_50_ of 5.7 μM, ZPT potentiates swelling-induced VRAC currents after the currents have reached a steady state and activates currents in the absence of cell swelling. ZPT is the first discovered small molecule VRAC activator, and the study of its mechanism will help improve the understanding of the VRAC pathway [15]. 

SCO-101(endovion) was initially developed to treat patients with stickle cell anemia 20 years ago, but the drug development was halted in 2003 because the four phase 1 trials found a dose-dependent reversible increase in plasma unconjugated bilirubin. However, in recent years, the interest in SCO-101 was rekindled because it was shown to inhibit the proliferation/migration of cancer cells, and it might be efficacious as an inhibitor of specific anticancer drugs. SCO-101 is now in its first phase 2 clinical trial, it may be a novel potential anticancer drug in the near future, However, there are currently no relevant clinical studies on its effect in cardiovascular diseases [16].

## 3. The Role of VRAC in Cardiovascular Diseases

The marked changes in the densities and/or properties of myocardial VRAC observed in association with CVDs, including arrhythmia, ischemic, remodeling myocardium and vascular diseases, are described below.

### 3.1. VRAC and Arrhythmia

The dysfunction of ion transporters, channels and their associated proteins is central to most inherited and acquired arrhythmia; a number of studies have firmly established the role of alternation in channel trafficking in arrhythmia [17]. In cardiomyocytes, while the electrogenic systems are mainly composed of sodium and potassium channels, there are also chloride channels that are necessary but should not be ignored. Scientists have found that chloride currents, such as the cAMP-dependent protein kinase A currents, CaCCs and VRAC, could modify cardiac electrical activity by contributing to actioning the potential abbreviation as well as resting membrane potential depolarization, thereby increasing susceptibility to arrhythmia. *I*_Cl_ are activated when the membrane potential is positive, and the increase of external Cl^−^ current accelerates the repolarization of myocardial cells. They are inhibited when cardiomyocytes are at resting membrane potential, resulting in an increase in inward Cl^−^ current, leading to the depolarization of cardiomyocytes. The activation of *I*_Cl,swell_ contributes to a mild depolarization of resting potential and shortened action potential duration, and slow down the conduction velocity of the myocardium, thus inducing the formation of reentry activity. In 2019, Yuriy and colleagues identified caveolae-mediated activation of mechanosensitive *I*_Cl,swell_ as a critical cause of the triggering impulses that can initiate atrial arrhythmia [18].

**Figure 1:**
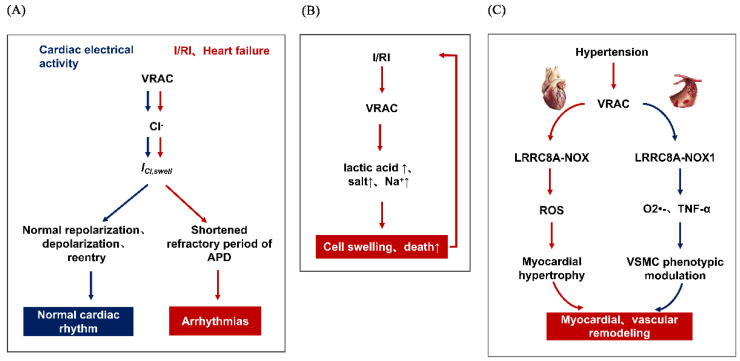
The role of VRAC in several cardiovascular diseases, (**A**) VRAC and arrhythmias, the blue part represents normal electrical activity of the heart, (**B**) VRAC and myocardial ischemic disease, (**C**) VRAC and cardiovascular remodeling, including the myocardial remodeling(red part) and vascular remodeling(blue part).

In addition, it has been reported that the continuous activation of *I*_Cl,swell_ is involved in the occurrence and development of arrhythmia caused by various pathological states, including the circumstance of ischemia-reperfusion injury (I/RI) and heart failure. This may be after acute and transient myocardial ischemia, the metabolism and membrane transport functions of myocardial cells are impaired, resulting in the accumulation of a large number of intracellular metabolites and a sharp increase in osmotic pressure, which results in extracellular water diversion into cells and cell swelling, and this situation will worsen sharply after myocardial reperfusion. Increased extracellular Cl^−^ concentration is a main risk factor of arrhythmia after I/RI. Ridley found that replacing Cl^−^ with NO_3_^−^ could significantly reduce arrhythmia caused by I/RI [19]. Both DCPIB, and DIDS and 4-acetamido-4-isothiocyanostilbene-2,2-disulfonate (SITS), can prolong the refractory period of APD and myocardium, thus playing an anti-arrhythmic role and preventing the occurrence of abnormal arrhythmia [20]. Shi et al. revealed, in 2021, that VRACs play an important role in the vulnerability to atrial fibrillation in dilated atria with heart failure and could be a potential therapeutic target for atrial fibrillation [21]. Thus, manipulation of VRACs may identify a novel therapeutic target for arrhythmia (Figure 1A).

### 3.2. VRAC and Myocardial Ischemic Disease

Cardiac cell swelling was observed after acute regional myocardial ischemia or ischemia-reperfusion induced injury (I/RI), therefore, swelling-induced cardiac VRAC activation is possibly involved in the process. It has been confirmed that the activation of the VRAC in I/RI is necessary for the following reasons: (1) the accumulation of anaerobic metabolites in the myocardium, and a large number of intracellular metabolites and waste, such as lactic acid and salt, leads to a significant increase in extracellular osmotic pressure, resulting in cell swelling; (2) hypoxia can also lead to the failure of intracellular Na^+^-K^+^ pump, resulting in a significant increase in intracellular Na^+^ concentration, resulting in aggravated cell swelling; (3) during myocardial reperfusion, extracellular permeable substances can be removed quickly, which further increases intracellular osmotic pressure and aggravates cell swelling, ultimately leading to the activation of VRAC. Due to the dual role of VRACs in Cl^−^ transport and ATP and glutathione release, the VRAC is considered to be a “double-edged sword” in the process of myocardial I/RI injury [22].

Actually, in 1999, a VRAC blocker IAA-94 was found to suppress the protective effect of ischemic condition with inhibiting the RVD response [23]. Wang et al. suggested that in mouse cardiomyocytes, VRAC blockers including DIDS, NPPB and phloretin could inhibit the apoptosis induced by ischemia reperfusion, thus increasing the activity of caspase-3, reducing the range of myocardial infarction, and promoting the recovery of cardiac function [24]. This is consistent with the report of Mizoguchi et al. [25]. However, contrary arguments have also been reported: Diaz et al. believed that NPPB and IAA-94 led to the expansion of the scope of myocardial infarction and increased the number and proportion of apoptosis in a rabbit I/RI model [26]. Due to the lack of specificity of these blockers, it is difficult to explain the role that the VRAC plays in the occurrence of cardiac I/RI; although reports vary, they all indicate that the VRAC was involved in the I/RI process (Figure 1B). 

### 3.3. VRAC and Myocardial Remodeling 

Myocardial remodeling occurs under the infrastructure of myocardial hypertrophy and fibrosis, which is often subsequently accompanied with heart failure progression. Cardiac hypertrophy defined as ventricular thickening and enlargement of the heart, caused by the increase in the volume of cardiac myocytes after the heart responds to volume or pressure load. Promisingly, as the most important anion channel in regulating cell volume, studies have revealed that the VRAC may be active during myocardial hypertrophy, and an increasing body of evidence suggests it is important in the process of adaptive remodeling in the failing heart [27]. In 2011, Yamamoto’s study suggested that the VRAC was involved in the process of cardiomyocyte hypertrophy by regulating cell volume for the first time, thus finding that the VRAC may be an important protective target of cardiac hypertrophy. In 2021, a new study of our group further elucidated that in the angiotensin II-induced cardiac hypertrophy model, the VRAC’s core structural protein LRRC8A interacts with NADPH through its LRRD domain to participate in the regulation of cardiac hypertrophy, knockdown of LRRC8A-attenuated AngII-induced cardiomyocyte hypertrophy [28]. Clemo et al. pointed out in the model of canine congestive heart failure caused by tachycardia that hypertrophic cardiomyocytes had a volume sensitive, outward-recirculating Cl^−^ current, which was continuously activated [29]. In addition, Chen et al. demonstrated, in 2019, that LRRC8A is upregulated in cardiac fibrosis following myocardial infarction (MI), and the cardiac-specific LRRC8A knockdown ameliorates the post-MI cardiac fibrosis and ventricular dysfunction via the JAK2-STAT3 signaling pathway [30]. Taken together, targeting the VRAC and LRRC8A may be a promising and effective therapeutic strategy for cardiac remodeling (Figure 1C).

### 3.4. VRAC and Vascular Disease

The series of structural and functional abnormalities caused by changes in the internal and external environment of blood vessels are called vascular remodeling, which is the most significant pathological feature of hypertension and an important pathological basis for the structural and functional abnormalities of heart, brain, kidney and other organs. At present, a large number of studies have confirmed that vascular remodeling may be related to hemodynamics, vasoactive substances, neurohumoral factors and other factors, but there are still many problems to be further clarified. In 2021, Li et al. found that in the process of hypertensive cerebrovascular remodeling, the VRAC structural protein LRRC8A promoted the proliferation of rat cerebral vascular smooth muscle cells by phosphorylation with no lysine (K) 1 (WNK1) [31]. Choi had investigated that in vascular smooth muscle cells, LRRC8A interacted with NADPH oxidase 1 (NOX1) to promote the production of extracellular superoxide anion O2•^−^ and the endocytosis of tumor necrosis factor-α (TNF-α), thereby promoting the proliferation and inflammatory response of vascular smooth muscle cells [32]. Meanwhile, the latest research results of Ahmad et al. suggest that LRRC8A plays an important role in maintaining vascular function by regulating the Akt-eNOS signaling pathway in vascular endothelial cells, and knockout of LRRC8A can lead to insulin resistance and damage vascular function by interfering with the PI3K-Akt-eNOS pathway [33]. The previous studies’ results point to a critical involvement of sphingosine-1-phosphate(S1P) in the pathogenesis of hypertension, S1P generated enzyme type 2 (SphK2) and S1P receptor1 (S1PR1) evolve as key players in immune trafficking and vascular dysfunction contributing to the development of vascular remodeling [34]. At the same time, Zahiri concluded that during stimulation with S1P, the BV-2 microglia cells secrete ATP via the VRAC, thus stimulating cell migration [35], Philipp et al. suggested that S1P-induced ATP secretion via the activation of the VRAC in macrophages constituted a functional process of macrophage migration [36]. In addition, the VRAC may alter endothelial cell membrane potential by regulating other ion channels in endothelial cells such as Piezol1, TRPV4 and downstream calcium signaling [37]. These data are the first to provide a molecular mechanism for the potent antiproliferative and anti-inflammatory effects of VRAC inhibition. One study demonstrated that volume-regulated Cl^−^ movement via VRAC was augmented during macrophage-derived foam cell formation, and its increment positively correlated with the intracellular cholesterol content as well as the atherosclerotic plaque area (Figure 1C) [38]. 

## 4. Conclusions

The VRAC is an important participant and regulator in the occurrence and development of CVD, and is closely related to the occurrence and development of several CVDs. Although the role of the VRAC in CVD is becoming increasingly understood, it is still lagging behind when compared with cationic channels. In addition, there is a lack of robust clinical trials to prove the effects of the VRAC, which would be of great value in revealing the role and mechanism of VRAC in CVDs. Therefore, investigations into VRACs and CVDs need to be further studied in order to provide credible and effective scientific bases for the future treatment of CVDs.

## Data Availability

No publicly archived data was established.
